# The Association of Female Reproductive Factors with Glaucoma and Related Traits

**DOI:** 10.1016/j.ogla.2022.06.003

**Published:** 2022-06-09

**Authors:** Kian M. Madjedi, Kelsey V. Stuart, Sharon Y.L. Chua, Paul J. Foster, Nicholas G. Strouthidis, Robert N. Luben, Alasdair N. Warwick, Jae H. Kang, Janey L. Wiggs, Louis R. Pasquale, Anthony P. Khawaja

**Affiliations:** 1 National Institute for Health Research, Biomedical Research Centre, Moorfields Eye Hospital NHS Foundation Trust & University College London, Institute of Ophthalmology, London, United Kingdom.; 2 Department of Ophthalmology, University of Calgary, Alberta, Canada.; 3 Medical Research Council, Epidemiology Unit, University of Cambridge, Cambridge, United Kingdom.; 4 UCL Institute of Cardiovascular Science, London, United Kingdom.; 5 Channing Division of Network Medicine, Brigham and Women’s Hospital, Harvard Medical School, Boston, Massachusetts.; 6 Department of Ophthalmology, Harvard Medical School, Massachusetts Eye and Ear Infirmary Boston, Massachusetts.; 7 Department of Ophthalmology, Icahn School of Medicine at Mount Sinai, New York, New York.

**Keywords:** Estrogen, Glaucoma, HRT, IOP, POAG

## Abstract

**Topic:**

This systematic review summarizes evidence for associations between female reproductive factors (age at menarche, parity, oral contraceptive [OC] use, age at menopause, and postmenopausal hormone [PMH] use) and intraocular pressure (IOP) or open-angle glaucoma (OAG).

**Clinical Relevance:**

Understanding the associations between female reproductive factors and glaucoma may shed light on the disease pathogenesis and aid clinical prediction and personalized treatment strategies. Importantly, some factors are modifiable, which may lead to new therapies.

**Methods:**

Two reviewers independently extracted articles in MEDLINE, Embase, Cochrane Database of Systematic Reviews, and Cochrane Central Register of Controlled Trials databases to identify relevant studies. Eligibility criteria included studies with human subjects aged > 18 years; a measured outcome of either IOP or OAG; a cohort, case-control, cross-sectional, or randomized controlled trial design; a reported measure of association, such as the hazard ratio, relative risk, odds ratio, or mean difference, with an associated confidence interval; and a measured exposure of at least 1 of the following variables: age at menarche, parity, OC use, age at menopause, or PMH use.

**Results:**

We included a total of 27 studies. Substantial differences in study designs, exposure and treatment levels, treatment durations, and variable reporting precluded a meaningful quantitative synthesis of the identified studies. Overall, relatively consistent associations between PMH use and a lower IOP were identified. Estrogen-only PMH use may be associated with lower OAG risk, which may be modified by race. No significant associations were found with combined estrogen-and-progesterone PMH use. No strong associations between parity or age at menarche and glaucoma were found, but a younger age at menopause was associated with an increased glaucoma risk, and adverse associations were identified with a longer duration of OC use, though no overall association with OC use was found.

**Conclusions:**

The association between PMH use and lower IOP or OAG risk is a potentially clinically relevant and modifiable risk factor and should be investigated further, although this needs to be interpreted in the context of a high risk of bias across included studies. Future research should examine associations with IOP specifically and how the relationship between genetic factors and OAG risks may be influenced by female reproductive factors.

Primary open-angle glaucoma (POAG) is the leading cause of irreversible blindness worldwide, with a global prevalence expected to reach 112 million by 2040.^1^ The pathogenesis of POAG is not fully understood, but the final common pathway for all proposed etiologies is optic nerve head damage with death of retinal ganglion cells (RGCs).^2^ A proposed mechanic etiology suggests that mechanical stress from increased intraocular pressure (IOP) may lead to this damage,^2^ whereas a vascular hypothesis posits that changes in vascular autoregulation may decrease blood flow to the optic nerve, leading to glaucomatous optic neuropathy.^2,3^

Estrogen may play a potentially neuroprotective role in glaucoma, and estrogen deficiency in particular is thought to contribute to glaucomatous damage via both mechanic and vascular mechanisms.^4–7^ Aqueous production and outflow pathways may be affected or modulated by estrogen,^4,8^ and administration of supplemental or exogenous estrogen has been shown to prevent RGC loss in animal models with high IOP.^9^ Supporting the vascular hypothesis, animal and clinical studies have demonstrated that estrogen upregulates the activity of nitric oxide synthase,^10^ an enzyme that mediates vascular tone and increases optic nerve perfusion.^9,11,12^ Furthermore, RGCs express estrogen receptors,^12^ and genetic studies have demonstrated that estrogen receptor polymorphisms are important in POAG pathogenesis.^13^

A number of epidemiologic studies provide further support for the potential associations between estrogen-associated female reproductive factors and glaucoma.^8,14–21^ Surrogate measures for reduced lifetime estrogen exposure, such as an earlier age at menopause^16,21^ and a later age at menarche,^20^ have been associated with higher glaucoma risks, but results are conflicting^14,19,22–24^ and high-quality human studies on IOP are lacking. Clinical studies have also found that exogenous estrogen supplementation (in the form of hormone therapy) for individuals in hypoestrogenic states such as menopause may be associated with a lower risk of POAG^18^ and lower IOP values,^4,25–30^ although the specific mechanism is unclear.

This systematic review summarizes the associations between female reproductive factors (as defined by the surrogate variables of parity, age at menarche, oral contraceptive [OC] use, age at menopause, reproductive duration, and postmenopausal hormone [PMH] use) with IOP and open-angle glaucoma (OAG; including POAG). Clarifying the associations between female reproductive factors and glaucoma (and related traits) may provide a better understanding of the sex-specific risk factors for OAG and may serve as a foundation for future preventative and therapeutic strategies in its management.

## Methods

We conducted a systematic review to assess and summarize the associations between key female reproductive factors (specifically, age at menarche, parity, OC use, age at menopause, duration of reproductive years, and PMH use) and IOP or OAG in accordance with the Preferred Reporting Items for Systematic Reviews and Meta-analyses guidelines.^31^ No predefined protocol for this systematic review has been published previously. As this study involved only a review and synthesis of existing literature, it was exempt from institutional review board approval. All research adhered to the principles outlined in the Declaration of Helsinki.

### Search Methods for Identifying Studies

We conducted a systematic search of the MEDLINE, Embase, Cochrane Database of Systematic Reviews, and Cochrane Central Register of Controlled Trials databases from inception to September 15, 2020, using keywords and medical subject heading terms relating to prespecified female reproductive factors and IOP and OAG. The search was conducted without restrictions on the time period, publication type, or language of publication. The detailed search strategies for MEDLINE, Embase, and the Cochrane Central Register of Controlled Trials are provided in Appendix SA1 (available at www.ophthalmologyglaucoma.org). We also performed a search of the Google Scholar database, hand-searched the reference lists of all included studies and relevant review articles, and contacted information specialists and clinical experts to identify potential additional studies not captured by our original database searches.

### Eligibility Criteria for Considering Studies for this Review

For inclusion into the systematic review, a study needed to meet the following criteria: (1) study participants were aged >18 years; (2) the study measured and reports at least 1 of the following female reproductive factors: age at menarche, parity, gravidity, OC use, age at menopause, duration of reproductive years, or PMH use; (3) the study reports either or both of the outcomes of IOP (measured in mmHg) or OAG, confirmed by an ophthalmic assessment, review of a previous assessment reporting structural or functional findings consistent with OAG (i.e., visual field assessment, optic nerve structural evaluation, or gonioscopy), or record linkage to a previously documented diagnosis of OAG (case ascertainment criteria in included studies are described in Table S1, available at www.ophthalmologyglaucoma.org); (4) the study employs a cohort, case-control, cross-sectional, before-and-after analysis, or randomized controlled trial (RCT) design; and (5) the study reports measures of association, including the hazard ratio (HR), relative risk, odds ratio (OR), or mean difference, with associated confidence intervals (CIs) or standard errors, or sufficient data are provided to allow for calculation of these measures. We excluded studies that (1) were review articles, animal studies, case reports, conference presentations, conference abstracts, letters to the editor, or basic science studies (although the latter were evaluated for background in the context of providing supporting evidence for the observational human studies summarized in the Results section); (2) did not specifically report either IOP or OAG data as an outcome; (3) did not perform an ophthalmic examination or use findings from a previous examination to confirm the OAG diagnosis; or (4) were published in a non-English language.

### Study Selection

Title and abstract screening was performed independently by 2 reviewers (K.M.M. and S.Y.L.C.) to identify studies relevant to the associations between female reproductive factors and IOP or OAG. All titles and abstracts were imported into an Excel (Microsoft Corporation) spreadsheet to facilitate review. Studies unrelated to the exposures or outcomes of interest (as well as duplicate studies) were identified by hand and excluded at this stage. The full text of the remaining studies was then retrieved to assess the study methods and data, and studies not fulfilling all the inclusion criteria at this point were excluded (Table S2, available at www.ophthalmologyglaucoma.org). Each reviewer independently extracted study results, which were exported as .RIS or .XLSX files into independent Excel spreadsheet forms, which were then consolidated. Discrepancies between reviewers were resolved by discussion between the 2 reviewers to achieve consensus and by consultation with a third reviewer (A.P.K.) if necessary.

### Data Collection and Risk-of-Bias Assessment

The following data were extracted from included studies: author names, year of publication, country, ethnicity of the study population, study design, number of subjects analyzed, number of eyes analyzed, determination or measurement of the specific female reproductive factor of interest and its levels, reference or control group, mean ages (for each group), specific outcome assessed (IOP, OAG), case definition of OAG, the most fully adjusted effect estimate for the association (OR, HR, other risk ratio, or mean difference), and its corresponding CI. We also collected the adjusted covariables for each fully adjusted effect estimate. Data were extracted only from the available published articles: authors of included studies were not contacted for missing or additional information. A formal risk-of-bias assessment was conducted at both the domain level and the individual study level, modeled on the Cochrane Collaboration’s Risk-of-Bias tool^32^ for randomized studies and the Risk of Bias in Nonrandomized Studies of Interventions^33^ tool for cohort, pre/post, and cross-sectional studies. The risk-of-bias domains assessed included the following: confounding, participant selection, measurement, or classification of the exposure; departures from the intended exposure; measurement or ascertainment of the outcome; reported outcomes; and missing data. Cases of disagreement in assessments of the risk of bias between the reviewers were resolved by discussion to achieve consensus and with consultation with a third reviewer (A.P.K.) if needed.

### Data Synthesis and Analysis

The extracted data from all included studies were arranged both by exposure (i.e., parity, age at menarche, OC use, age at menopause, duration of reproductive years, and PMH use) and subgroup where possible (i.e., estrogen-only PMH and estrogen-plus-progesterone PMH use, age at natural menopause, and age at surgical menopause). We then assessed whether the data were suitable for quantitative summarization (i.e., meta-analysis). Although no formal statistical tests of heterogeneity were calculated, substantial differences were found across study designs, exposure levels, treatment doses, and treatment durations, and it was noted that different studies measuring a given exposure frequently used different exposure levels as a reference. A consensus between authors determined these factors precluded a meaningful meta-analysis.

## Results

**Figure 1.**
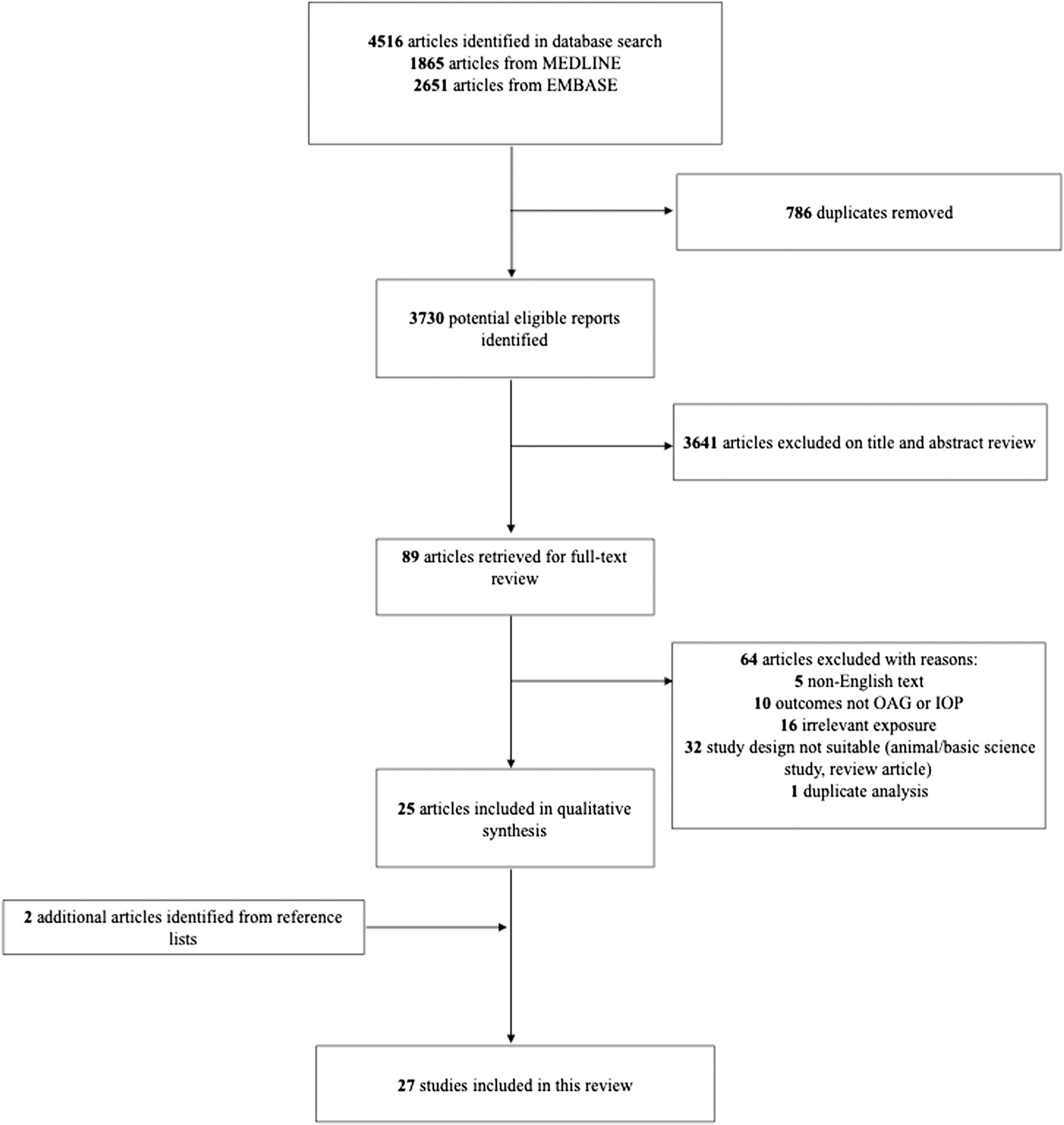
Flow diagram describing the process of study selection in line with the Preferred Reporting Items for Systematic Reviews and Meta-analyses. IOP = intraocular pressure; OAG = open-angle glaucoma.

A total of 4516 articles were initially identified. After removal of duplicates, 3730 articles remained, which underwent title and abstract screening by 2 authors (K.M.M. and S.Y.L.C.). The full texts of 89 articles were retrieved for review. Of these articles, 5 were non-English; 8 reported outcomes irrelevant to this review; 17 examined irrelevant exposures; 33 were either review articles, animal studies, or basic science studies; and 1 was a duplicate study, and these were excluded. A careful examination of the reference lists of the included articles identified 2 additional relevant studies that met the inclusion criteria and were ultimately included in this review for a total of 27 studies. Characteristics of the included studies are described in Tables 1 and 2.^4,6,8,14–19,21–30,34–41^ Figure 1 describes the full identification, screening, and inclusion process according to Preferred Reporting Items for Systematic Reviews and Meta-analyses guidelines.

### Risk of Bias

**Figure 2.**
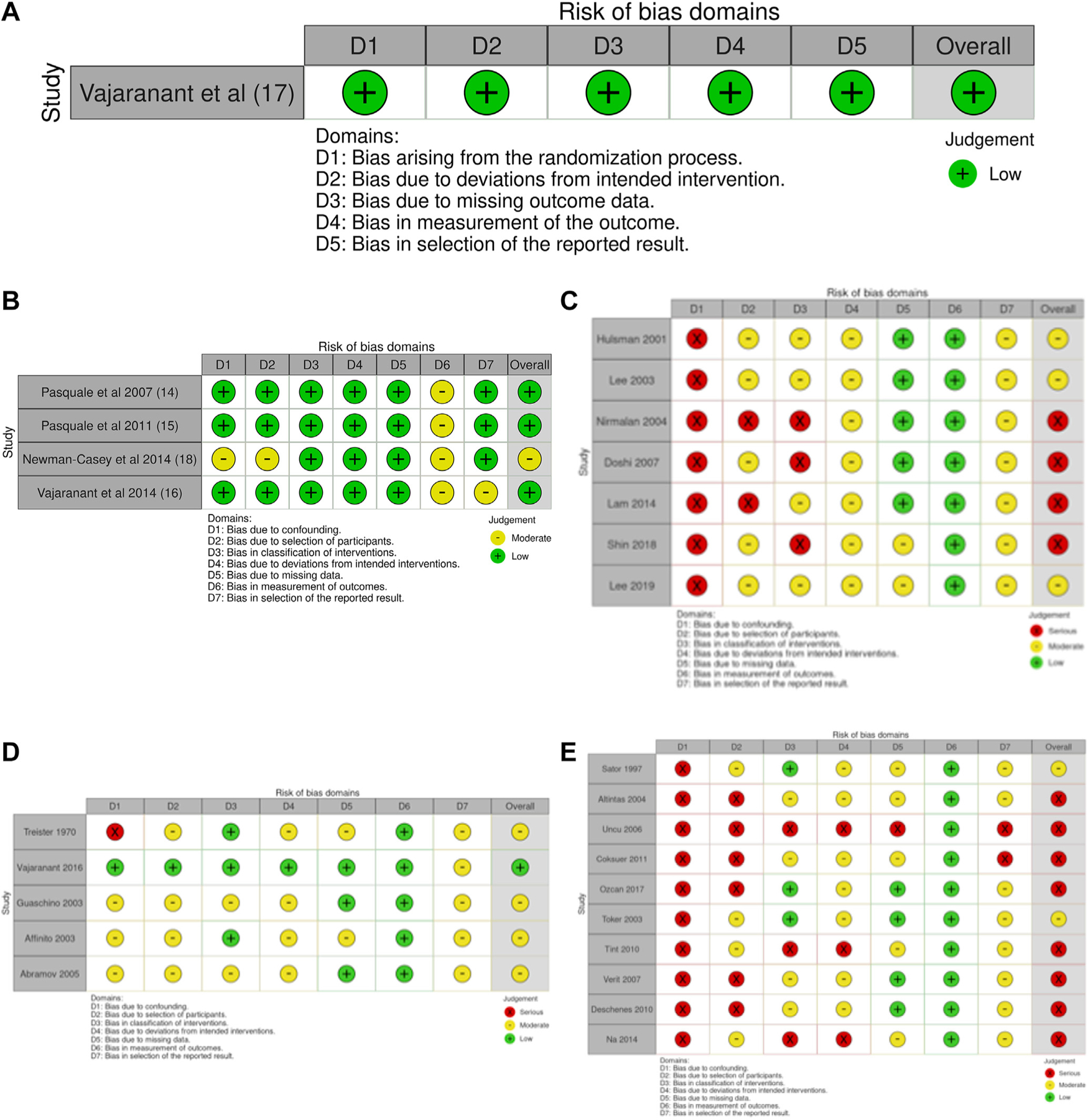
**A,** Risk-of-bias assessment of randomized controlled trials included in the systematic review of female reproductive factors and open-angle glaucoma. *Secondary data analysis from a randomized controlled trial. **B,** Risk-of-bias assessment of cohort studies included in the systematic review of female reproductive factors and open-angle glaucoma. **C,** Risk-of-bias assessment of cross-sectional studies included in the systematic review of female reproductive factors and open-angle glaucoma. **D,** Risk-of-bias assessment of controlled studies included in the systematic review of female reproductive factors and intraocular pressure. **E,** Risk-of-bias assessment of uncontrolled studies included in the systematic review of female reproductive factors and intraocular pressure.

This review includes studies with different designs and therefore required the use of > 1 risk-of-bias tool. Sources of bias varied according to the study design, outcome, and risk-of-bias tool used and, as such, were not directly comparable. Results of the risk-of-bias assessment are presented separately based on the study design and outcome of interest (Fig 2). The formal risk-of-bias assessments identified that confounding, participant selection, and exposure classification represented the most significant sources of bias. The risks of bias for studies using OAG as an outcome ranged from low to serious and differed based on the included study design. The risks of bias for RCTs and cohort studies examining the associations with OAG were low, with the exposure classification representing the greatest source of bias. The risks of bias for cross-sectional studies assessing the associations with OAG were serious overall, with substantial risks of bias from the confounding, participant-selection, and exposure-classification domains. When studies on OAG with moderate to serious risks of bias were excluded, the remaining studies with a low risk of bias (including RCTs and prospective cohort studies) identified consistent relationships whereby age at menopause and PMH use were associated with lower risks of OAG.

For controlled studies with IOP as the outcome, the overall risk of bias was moderate, and it was serious for uncontrolled studies on IOP. This was particularly influenced by a serious risk of bias in the confounding domain. When studies on IOP with a moderate or serious risk of bias were excluded, the remaining studies with a low risk of bias (an ancillary analysis of an RCT and a prospective cohort study) identified that participants using an estrogen-only PMH had significantly lower IOP values compared with placebo controls and a lower risk of high-tension POAG (i.e., IOP > 21 mmHg). The overall risk of bias was moderate for associations with OAG and high for associations with IOP.

### Age at Menarche

Five included studies examined the association between the age at menarche and risks of OAG.^15,19,22–24^ Four of these studies were cross-sectional in design^19,22–24^ and included a total of 14 294 individuals with 560 OAG cases, and 1 study^15^ was a prospective cohort study describing 808 incident POAG cases over 1 288 200 person-years of follow-up (Table 3).

Undergoing menarche at a later age (i.e., between 13 and 14 years) was associated with twice the risk of POAG (OR, 2.1; 95% CI, 1.1–3.8) compared with achieving menarche at an age < 13 years in 1 study,^24^ but no other associations were identified across the included studies. The overall preponderance of observational studies does not support an association between age at menarche and OAG.

### OC Use

Four included studies explored the association between OC use and OAG risks. Three were cross-sectional studies^19,24,34^ that included 487 cases from 2573 subjects, and 1 was a cohort study of 333 POAG cases observed over 619 356 person-years of follow-up^15^ (Table 4). Data from the Nurses’ Health Study (NHS)^15^ found no overall association between ever using OC and incident POAG but identified a trend between a longer duration of OC use and higher POAG risks (*P* for trend = 0.04), with prolonged use of OC (> 5 years) associated with a 25% higher risk (relative risk, 1.25; 95% CI, 1.02–1.53). Other studies evaluating the relationship between any use of or ever using OC and OAG did not identify any overall association.^19,24,34^

### Parity

Five included studies reported the association between the number of pregnancies (parity) and OAG risks.^15,19,23,24,35^ Four of these^19,23,24,35^ were cross-sectional (a total 601 cases from 13 078 participants) and 1 study^15^ was a prospective cohort study with a total of 806 incident POAG cases discovered during 1 280 918 person-years of follow-up (Table 5). Different reference groups were used in each study, which precluded a direct, quantitative summarization. Individuals with 5 or more pregnancies had 2.5 times the risk of OAG compared with nulliparous women (OR, 2.5; 95% CI, 1.1–6.1) in 1 cross-sectional study,^24^ and women with 3 or 4 children had twice the risk of OAG compared with women with only 2 children (OR, 2.1; 95% CI, 1.1–3.9) in a cross-sectional population study in Korea.^35^ A significant trend between an increasing number of pregnancies and an increased risk of OAG was identified in another study^24^ (*P* for trend = 0.03), but no association was reported in the other 2 included studies.^15,23^ The association was inconsistent across the included studies, and results from the most highly powered study suggested no direct association between parity and OAG risks.^15^

### Age at Menopause

Seven included studies investigated the association between age at menopause and risks of OAG.^14,16,19,21–24^ Five of these studies were cross-sectional in design (with a total of 9501 individuals with a total of 259 cases of OAG),^19,21–24^ and 2 were cohort studies^14,16^ with a total 462 cases over a combined 687 020 person-years of follow-up (Table 6).

The cohort studies found that women with earlier menopause had a 1.6-times higher risk of all-cause glaucoma (OR, 1.6; 95% CI, 1.5–2.2),^16^ although the association was not significant for OAG specifically. The cross-sectional studies reported associations with age at menopause and OAG, finding that an earlier age at menopause was associated with higher odds of OAG of 2.3 (95% CI, 1.2–45)^19^ and 3.5 (95% CI, 1.2–10.1).^21^

Among studies reporting results for all-cause menopause (i.e., both natural and surgical cases combined), 1 study found that women undergoing earlier menopause (before 53 years of age) had a significantly higher risk of OAG than those who underwent menopause after the age of 53 (OR, 3.4; 95% CI, 1.2–9.8).^21^ When the analysis was limited only to women with a reported age at natural menopause, earlier menopause was associated with more than twice the risk of OAG in 3 of the included studies.^19,21,22^ When stratified on chronologic age, older postmenopausal women (over age of 65) who underwent menopause later than age 54 had nearly half the risk of POAG of similar-aged women with earlier menopause in a secondary analysis of the NHS cohort.^14^ No significant association between OAG with a history of elevated IOP (> 21 mmHg) and age at menopause was identified in the 2 studies examining that specific relationship^14,22^ (Table 7). Overall, the direction and consistency of findings suggest an association between a younger age at menopause and higher risks of OAG, with associations identified relatively consistently across the included studies, supported by subanalyses in large cohort studies.

### Years of Reproductive Duration

Four studies (all previously described) provided specific data on the total number of years of reproductive duration (i.e., duration between ages at menarche and menopause) and risks of POAG^15,22–24^ (Table 8). Each additional year of reproductive duration was associated with a 5% reduction in the odds of OAG in an Australian study^24^ (OR, 0.95; 95% CI, 0.90–0.99), but no association was identified in other cross-sectional studies or in the NHS, which was the largest and most highly powered study of this association.^14^

### PMH Use and OAG Risks

We identified 7 studies examining PMH use and risks of OAG. One study was an ancillary study within a placebo-controlled RCT,^17^ 2 studies^14,18^ were cohort studies (Table 9), and 4 studies^19,22,24,34^ were cross-sectional population studies involving a total of 18 152 participants with 853 cases of OAG (Table 10).

The ancillary analysis of the Women’s Health Initiative Sight Exam Study RCT and the 2 cohort studies provided individual analyses for OAG risks associated with the specific type of PMH used (i.e., estrogen-only or combined estrogen plus progesterone) and, as such, are discussed separately from the cross-sectional studies that examined the use of unspecified preparations of PMHs as the main exposure of interest.

Among the RCT and cohort studies examining different formulations of PMHs, estrogen-only PMH use was examined in a total 1412 individuals in 3 studies (Table 9). Estrogen-only PMH use was associated with a 0.4% decreased risk of POAG per additional month of use (HR, 0.996; 95% CI, 0.993–0.999) in 1 cohort study of health insurance claims in a US-based population.^18^ No overall association was found between estrogen-only PMH use and OAG risks in the Women’s Health Initiative Sight Exam Study trial (HR, 1.01; 95% CI, 0.79–1.29), although a prespecified analysis found the risk of incident OAG to be lower in Black women using an estrogen-only PMH compared with placebo (HR, 0.49; 95% CI, 0.27–0.88; *P* for interaction = 0.01), and this association was not identified in White participants.^17^

No overall association between combined estrogen-and-progesterone PMH use and OAG risks was identified in any of these 3 studies,^14,17,18^ although a subgroup analysis of the NHS data found that current use of a combined PMH was associated with a slightly lower risk of high-tension POAG (defined as IOP > 21 mmHg).^14^ There was no significant association between PMH use and OAG in the 4 cross-sectional studies.

### PMH Use and IOP

Fifteen studies assessing the associations between PMH use and IOP were identified (Tables 11–13).^4,6,8,25–30,36–41^ Five studies were controlled studies^8,25,27,36,37^ (Table 11), 6 studies were uncontrolled cross-sectional studies^6,30,38–41^ (Table 12), and 4 were uncontrolled before-and-after studies measuring IOP values within participants at baseline and then following a period of PMH use.^4,26,28,42^

Controlled studies were further divided into those that reported analyses from estrogen-only PMH user groups and combined estrogen-plus-progesterone user groups (Table 11). A total of 823 eyes of individuals receiving an estrogen-only PMH were compared against a total of 875 control eyes of individuals receiving either a placebo control or no PMH. Estrogen-only PMH user groups had lower IOP values (−0.5 mmHg^8^ and −2.0 mmHg^25^) compared with controls in both studies. Four studies evaluated a combined estrogen-plus-progesterone PMH user group against a control group.^8,27,36,37^ A total of 1584 eyes of individuals receiving a combined estrogen-plus-progesterone PMH were compared against a total 1469 control eyes. Two studies identified lower IOP values in the combined PMH group than in the control group^25,27^ (14.1 ± 2.0 vs. 16.6 ± 2.4, respectively; −1.8 mmHg; 95% CI, −2.2 and −1.4 mmHg). The largest placebo-controlled RCT to date on the association between PMH use and IOP^8^ found the IOP values of eyes treated with the estrogen-only PMH to be lower by 0.5 mmHg (95% CI, −0.8 and −0.1 mmHg; *P* < 0.05) than eyes receiving placebo after 5 months of treatment, but no difference in IOP was found in participants using combined estrogen plus progesterone over the same period of time.^8^ Although this study reported the difference in IOP values between treated and placebo groups for both types of PMHs independently, it did not compare the relative effects of estrogen-only to estrogen-plus-progesterone treatment.

Across the 6 uncontrolled cross-sectional studies, 2 studies^40,41^ identified different IOP values between PMH users and nonusers, with 1 study identifying a 1.3-mmHg (11.9 ± 2.7 vs. 13.2 ± 2.9, respectively)^40^ lower IOP value in PMH users and 1 identifying a 0.4-mmHg^41^ (14.8 ± 0.1 vs. 14.4 ± 0.3, respectively) higher IOP value (Table 12). These studies did not specify the type of PMH used.

Four studies using an uncontrolled pre/post design were identified (Table 13).^4,26,28,29^ Three studies assessed IOP values within participants before and after a course of combined PMH therapy, and 1 study separately assessed IOP values in 2 groups (before and after a course of estrogen-only PMH therapy in 1 group and separately for combined PMH therapy in the other). None of these studies compared IOP between the groups in question. The single study evaluating estrogen-only PMH use reported a 2.0-mmHg lower IOP value within participants after PMH use.^26^ The remaining studies examining combined PMH use found participants to have overall lower IOP after PMH use, with differences ranging from −0.7 mmHg^28^ to −3.8 mmHg^4^ lower after a course of PMH therapy.

## Discussion

Evidence from the 27 included studies highlights potentially important associations of various female reproductive factors with glaucoma and IOP. Although associations between PMHs and IOP or POAG were consistent across these studies, modest associations with other factors, including the duration of OC use and age at menopause, were reported in several studies.

### Age at Menarche

A younger age at menarche should theoretically confer greater overall lifetime estrogen exposure, which would lead to a hypothetically lower risk of POAG. Evidence from the included observational studies,^14,19,22–24^ however, suggests no clear association between the age at menarche and risks of POAG. This may be owing to the inability to meta-analyze the various studies, leading to this review being underpowered to identify a true association. Although no studies directly examined the association between age at menarche and IOP, a secondary analysis of the NHS found that a later age of menarche was associated with a slightly higher risk of the normal-tension subtype of POAG (IOP < 22 mmHg),^14^ suggesting that a potential association between menarche age and glaucoma may occur via non–IOP-mediated mechanisms. The relationship between age at menarche and POAG should be further investigated, more completely accounting for the entire female reproductive and postreproductive history.

### Parity

Evidence from observational studies^15,19,23,24,35^ suggests there is likely no direct relationship between the number of term pregnancies and the risk of POAG. Although the Blue Mountains Eye Study^24^ and the Korean National Health and Nutrition Examination Survey (NHANES)^35^ reported higher numbers of pregnancies to be associated with higher risks of POAG, these results were not reproduced in other studies, and no consistent trend was found between increasing parity and increasing risks of POAG. Interestingly, no included study directly compared the risks of POAG between nulliparous women and women with any non-0 number of children, but 1 excluded study reported larger vertical neuroretinal rim widths in women of any parity than in nulliparous women, suggesting potential associations between parity and other glaucoma phenotypes.^43^

### OC Use

Although the existing literature does not support a direct association between OC use and glaucoma-related traits, potential adverse associations with the duration of use have been identified. The nature of the association between ever or current OC use and OAG remains incompletely studied, but results from the NHS provide support for a potential relationship between a longer duration of use and higher POAG risks in particular.^15^ This association has been further investigated in a recent, large, US-based study (Wang et al,^20^ excluded from this review), which similarly found a greater duration of OC use to be associated with a substantive increase in the odds of self-reported glaucoma and ocular hypertension compared with individuals with no history of OC use. As this study used self-reported glaucoma or ocular hypertension as outcomes, it was excluded from our systematic review, although it highlights the growing need for research around the potential associations between the duration of OC use and glaucoma and related traits. Interestingly, no associations have been identified with other glaucoma-related traits, such as visual field defects or an increased vertical cup-to-disc ratio, and OC use in secondary analyses of Wang et al^20^ and another excluded study.^43^

Decisions made by women around contraception use are influenced by numerous demographic, socioeconomic, and geographic factors,^44,45^ which may potentially explain or modify any potential associations between OC use and IOP or OAG. The inconsistent associations found among studies examining parity and OC use may be related to the age range during which OC use typically occurs versus the typically older age of glaucoma onset. This makes it challenging to identify strong and consistent associations across studies. Furthermore, no large-scale studies have been conducted examining OC use and IOP, and this remains a highly fertile area for further investigation.

### Age at Menopause

The epidemiologic literature does not consistently support an overall association between age at menopause and POAG; however, several subgroup analyses suggest a higher risk of POAG in those with an earlier age at natural menopause. A lower risk of POAG was also found in a large subgroup analysis of older women (> 65 years) who underwent menopause at a later age, suggesting that a longer duration of estrogen exposure may reduce the POAG risk.^14^ Although no association between the age at menopause and OAG with elevated IOP (specifically, > 21 mmHg) was identified, no study directly assessed the relationship with IOP, and this represents an avenue for future investigation. Such a study may, however, prove logistically challenging, as it would require measuring IOP values before and after the menopausal transition and adjusting for age.

Menopause can occur naturally or can be induced by surgery or radiation. Each of these types of menopause can influence the age at menopause,^46^ but the specific effects of each are not yet fully understood.^47^ The number of studies reporting each of these subtypes individually did not make a subanalysis realistic in this review, although an effort was made to do so. A recent genetic risk score of 18 single nucleotide polymorphisms strongly associated with the age at natural menopause (explaining 4.8% of the variation in age at natural menopause) was not found to be associated with POAG,^48^ suggesting no likely underlying genetic association for the findings from the included observational studies. Interestingly, 1 included study found that individuals who underwent surgically induced menopause (via bilateral oophorectomy) before the age of 43 had 1.6 times the risk of glaucoma (broadly defined) compared with a referent group of age-matched women who did not undergo oophorectomy (OR, 1.6; 95% CI, 1.15–2.23). The association with OAG specifically was not significant, although this particular analysis was likely underpowered.^16^ This study examined the youngest population of menopausal women (under age 43) and was the only study to specifically assess surgically induced (rather than natural) menopause. Although confounding by indication for surgery cannot be entirely ruled out, this study’s finding that menopause induced at an extremely early age may increase the risk of glaucoma lends further support to the role estrogen deficiency may play in glaucoma pathophysiology.

### Years of Reproductive Duration

Ages at menarche and menopause are surrogate variables for years of reproductive duration, a more direct (albeit imperfect) measure of endogenous estrogen exposure. Only 1 study^22^ identified a modestly lower risk of POAG from either a later age at menopause or an earlier age at menarche. The association between the duration of reproductive years and POAG risks remains unclear, and future assessments of the role of reproductive years in glaucoma pathogenesis may benefit from the development of genetic risk scores that account more accurately for endogenous estrogen exposure.

### PMH Use

Estrogen levels gradually decline throughout the reproductive years and following menopause. Postmenopausal women are, therefore, at a theoretically increased risk of glaucoma and related traits, and this risk would hypothetically be attenuated by the use of exogenous estrogen supplementation.

This review found that PMH use may be associated with lower IOP, but its impact on mitigating OAG risks remains unclear. One study found a significant association between PMH use and an overall decreased risk of POAG,^18^ whereas another cohort study found a decreased risk only for the high-tension (i.e., IOP >21 mmHg) subtype of POAG and another study reported no overall association but found race to be a significant modifier of the association.^17^ In this large study (a secondary analysis of an RCT), analyses stratified by race identified a significant risk reduction in OAG during the follow-up period among Black women using an estrogen-only PMH, but no association was identified in White women, suggesting race may modify the association between estrogen-only PMH use and OAG.^17^

In each of these studies, the association was only significant for users of an estrogen-only PMH. Multiple studies examined the association between PMHs and IOP, with most identifying an association between the use of PMHs and lower mean IOP values,^4,25–30,40,41^ and this association with lower IOP was more consistent among estrogen-only PMH users than among combined estrogen-plus-progesterone users.^8,25,26^ Any potential relationship between PMH use and lower risks of POAG might, therefore, be hypothesized to occur through an IOP-mediated pathway, potentially driven through estrogen-receptor signaling.

Although this review attempted a subgroup analysis of studies based on estrogen-only or estrogen-plus-progesterone use, the doses and durations of use differed between studies, precluding a meaningful direct comparison. Furthermore, several of the included studies assessing IOP did not perform comparisons against a formal control group and, as such, provide different effect estimates on the associations (or effects of the associations) with IOP than studies with a control group. Ultimately, observations in uncontrolled samples do not allow for the estimation of relative effects that would be necessary for decision-making.

Although there is variability in the quality of the included studies, the overall preponderance of evidence suggests a potential association between PMH use and lower IOP, and this warrants further investigation. Notably, evidence from RCTs and prospective studies suggests that PMH use may be associated with lower IOP, and this is further supported by similar results from other studies with less robust study designs. Although causality cannot be assessed, the association is consistent across included studies (and is consistent across studies with a low risk of bias), which strengthens the level of certainty for this potential association. These associations should be further explored in different ethnic groups, employing appropriate age- and race-matched reference groups receiving standardized doses of PMHs with similar follow-up periods. Future genetic epidemiologic studies may offer insight into race as a potential modifier of the association between estrogen-only PMH use and POAG and explore whether these relationships also apply to the association with IOP.

### Risk of Bias and Sources of Heterogeneity

Several important sources of heterogeneity and potential bias were identified across the included studies, which made a direct comparison of the findings challenging and ultimately precluded a formal statistical synthesis of the data. Different study designs were employed (including cross-sectional, longitudinal, and RCT designs), participants were included from different countries and ethnicities, and analyses did not always adjust for the same covariables.

Although most studies examining associations with OAG adjusted for age and often other socioeconomic or medical covariables, residual confounding from unmeasured covariables remains a possibility such that the identified associations may be driven by unknown or unmeasured factors. Several studies adjusted for a relatively comprehensive array of covariables, whereas others adjusted for fewer covariables (Tables 1 and 2). Several of these studies were uncontrolled before-and-after studies, which estimated different estimands than studies with a formal separate control group (observations in uncontrolled samples do not allow for estimation of relative effects). Collectively, these factors contribute to an overall serious risk of bias in the confounding domains for these studies in particular.

Exposure data were collected in different ways: most studies used self-reported data for female reproductive factors, whereas others used health records. Both of these approaches pose potential challenges for data integrity, as self-reported exposures can be associated with recall bias (especially with remote exposures such as OC use) and results from record use are directly tied to the accuracy of data entry in the first instance. This potential for bias in studies of PMH or OC use was offset in some studies by the use of prospective follow-ups confirming medication use, by linkage to pharmacy records of filled medication prescriptions, or by determining serum confirmation of estrogen levels. Exposure departures remain a distinct possibility, particularly in cross-sectional studies, but are less likely to be a substantial source of bias in the included cohort or before-and-after interventional studies because participants were either directly administered the treatment in question or had verified records confirming prescriptions were filled.

Importantly, there is also a degree of intrinsic variability within the exposures of interest themselves: women reach female reproductive health milestones at different ages^49,50^ that reportedly differ based on geography^51^ and may be modified by lifestyle factors and race.^52,53^ Furthermore, use of OCs and PMHs is influenced by various socioeconomic and demographic factors.^49,54,55^ Although no included studies assessed or reported interactions between various female reproductive factors of interest, at least 1 study adjusted for all the measured female reproductive factors in multivariable analyses.^15^ Potential interactions between female reproductive factors themselves and socioeconomic status (SES) represent an area for further investigation with regard to any potential associations with glaucoma or related traits.

Another major source of both heterogeneity and bias is the criteria used to define the outcomes of interest. Epidemiologic definitions of OAG in observational studies have varied over time,^56,57^ and this is reflected in different case definition criteria employed in the included studies (Appendix SA2). Although most included studies reported POAG as the main outcome and either excluded or adjusted for secondary OAGs (including pseudoexfoliation glaucoma), several did not specifically differentiate between POAG and other OAGs, and others included subjects with probable (but not necessarily definite) POAG (see Appendix SA2). Furthermore, most studies employed a direct physical examination to diagnose POAG, whereas other studies relied on previously conducted assessments. This variability in outcome definitions may have led to unintended biases, weakening our ability to identify potential associations. The studies examining IOP were more consistently robust in outcome ascertainment, as IOP was directly measured in each study. Intraocular pressure may be influenced by corneal biomechanical properties, and these were not directly measured or accounted for in any of the included studies. Nearly all studies used Goldmann Applanation Tonometry, although 2 studies used other methods to measure IOP (Table 2). Although this adds a degree of heterogeneity, the magnitude of any variability introduced in an outcome measurement is unlikely to bias our results in a substantial manner given the general concordance between noncontact tonometry and Goldmann Applanation Tonometry methods of IOP measurement.^58^

These sources of heterogeneity ultimately precluded quantitative summarization of the included studies, leading to this review being potentially underpowered to draw definitive associations between female reproductive factors and OAG. Despite these limitations, the overall trends of association are relatively consistent for certain reproductive factors (particularly PMH use, age at menopause) and are particularly consistent in studies with the lowest risks of bias. When interpreted collectively, these findings lend additional support to the hypothesis that estrogen-associated factors are associated with glaucoma and related traits.

### Strengths and Limitations

To our knowledge, no existing systematic review has consolidated all the studies examining the associations between a comprehensive set of female reproductive factors and both IOP and the OAG risk. This review addresses the existing gap in the literature and highlights areas for future research on this wide-ranging topic. The minimization of exclusion criteria during the search strategy helped ensure the review was highly comprehensive in its inclusion of relevant studies. Furthermore, the broad search criteria employed in the search strategy contributed to the comprehensiveness of this review in capturing multiple relevant exposures and outcomes.

Studies of female reproductive attributes in relation to OAG can be particularly useful, as controlling for age can help isolate the role female reproductive aging may play in the glaucoma disease processes. Female reproductive milestones that practically all women experience can be categorized by altered estrogen levels; by these proxy measures, one can examine how altered estrogen signaling may impact glaucoma more broadly. More directly, studying female reproductive milestones in relation to glaucoma or glaucoma endophenotypes can further inform our understanding of the pathophysiology of disease. In a recent study using a bioinformatics pipeline to prioritize the many newly discovered IOP-associated genes,^59^ estrogen-receptor signaling was a key modulator of several of these IOP-associated genes. These genetic associations further underscore the importance of better understanding the associations of female reproductive milestones with glaucoma and IOP.

The observational studies included in this review are susceptible to various biases, which prevent assessments of causal associations as discussed above. Epidemiologic assessments of female reproductive factors and their potential associations are complicated by a number of factors. Menarche and menopause, for example, are exposures that occur over short time frames in different relation to the typical onset of menopause, which poses a number of challenges for data collection and analysis. For the age at menarche, data collection is particularly challenging, as this female reproductive milestone is reached many decades before glaucoma typically occurs, whereas the age at menopause is typically reached 1 to 2 decades before the onset of glaucoma. These exposures are typically ascertained by recall and, if not accurately determined, may lead to misleading results for the relations between these exposures and the risk of glaucoma. Because these exposures have a small range of values and, probably, a modest effect on the disease process, very large sample sizes are necessary for studies to be appropriately powered to detect definitive associations with glaucoma.

As the risk for glaucoma increases with age,^60–62^ it is often challenging to distinguish the effects of female reproductive aging from those of chronologic aging on glaucoma and related traits. Furthermore, certain female reproductive factors (such as age at menopause^63^ and parity^64^) may be associated with socioeconomic factors, and economic deprivation may also be associated with higher rates of glaucoma or more advanced disease at initial presentation.^65,66^ Although several studies adjusted for SES, not every study assessed this in the same manner; thus, residual confounding by SES may potentially explain some of the above-identified observational associations.

Another major limitation of the included studies is that analyses were performed in predominantly White populations, limiting generalizability of these findings to other ethnic groups. This is particularly notable given that race has been identified as a possible modifier of the identified associations between PMHs and POAG.^17^

One limitation of many of the included studies examining POAG as the main outcome of interest is that profiling of the IOP values of the population at risk for glaucoma was not performed, which makes extrapolation of results to populations at risk somewhat more challenging. Given that the risk of developing OAG in a healthy population has been shown to increase by 16% per 1-mmHg increase in IOP,^67^ we hypothesize that factors that increase IOP in the general population will, in turn, increase the risk of OAG. This has been demonstrated to be the case in genetic associations: genetic determinants of IOP in general populations are also risk factors for OAG in independent case-control studies.^68^

Studies that examined reproductive factors but did not mention them in the title or abstract may have been missed, but we believe our search criteria to be reasonably robust. Several important studies were excluded from this paper for using self-reported glaucoma as the outcome of interest, despite providing evidence for potentially important associations of interest to this review. Similarly, several studies only provided data for OAG overall, which prevented the analysis of whether the identified associations are specific to POAG or may be associated with other phenotypes of OAG more generally. Another weakness of this review is its exclusion of non-English articles, which may give rise to a degree of publication bias, the extent of which was not formally assessed.

## Conclusions

The findings of this systematic review provide support for potential associations between certain female reproductive factors and IOP and OAG, although the reported associations need to be interpreted in the context of high risks of bias across included studies. A longer duration of OC use may be associated with a higher risk of OAG on the basis of included studies, but it is not yet clear whether use of OCs in general increases the risk of OAG or whether this potential association is mediated by IOP. An earlier age at natural menopause may be associated with a higher OAG risk. Most notably, PMH use may be associated with a lower IOP, and the estrogen-only type of PMH may be associated with a lower risk of OAG (a relationship which may be modified by race^17^). There is a clear need for additional studies on the associations between female reproductive factors and glaucoma and related traits. As the prevalence of OAG is expected to increase in the coming years, investigations into sex-specific risk factors and gene-environment interactions will be important in improving our understanding of the associations and pathogenesis of OAG, potentially leading to novel preventative measures and therapies.

## Supplementary Material

Table S2

Table S1

Appendix SA1

## Figures and Tables

**Table 1.** Characteristics of Studies Reporting an Association with Female Reproductive Factors and Open-Angle Glaucoma Included in the Systematic Review*

Author (Yr)	Location	Design	Total Women Participants, n	Mean Age of Overall Study Population, Yrs	Predominant Ethnicity of Study Population	Female Reproductive Factor Assessed	Adjustments
Randomized controlled studies							
Vajaranant et al (2018)^17,†^	USA	Post hoc, ancillary analysis of data from a randomized controlled trial	8102	69	White or Black	6	Age, concurrent dietary trial enrollment (race, age at menopause, diabetes, HTN, alcohol, smoking, BMI)^‡^
Cohort studies							
Newman-Casey et al (2014)^18^	USA	Retrospective cohort study using record linkage or historical data	125 163	65	White	6	Age, race, household net worth, region of residence, osteoporosis, retinal vascular occlusion, obesity, depression, diabetes, myocardial infarction, cataract, PDR, lens status; type of PMH used
Pasquale and Kang (2011)^15,§^	USA	Retrospective cohort	79 440	–	White	1, 2, 3, 5	Age, time at risk, family history of glaucoma, Black race, HTN, T2DM, smoking, alcohol intake, caffeine intake, BMI, physical activity, PMH use, age at menopause, age at menarche, OC use^‖^
Pasquale et al (2007)^14,§^	USA	Retrospective cohort study	58 144	–	White	4, 6	Age, time at risk, family history of glaucoma, Black race, HTN, T2DM, smoking, alcohol intake, caffeine intake, BMI, physical activity, PMH use, age at menopause, age at menarche, OC use^‖^
Vajaranant et al (2014)^16,¶^	USA	Age-matched, retrospective cohort	2114	–	White	4	Age, hypertension, obesity, diabetes, lipid metabolism disorders
Cross-sectional studies							
Hulsman et al (2001)^22,#^	The Netherlands	Population cross-sectional study	3078	69	White	1, 4, 5, 6	Age, diabetes status, HTN, PMH use, duration of PMH use
Lee et al (2003)^24,^**	Australia	Population cross-sectional study	2072	66	White	1, 2, 3, 4, 5, 6	Age, T2DM, HTN, myopia, PXG, family history of glaucoma
Nirmalan et al (2004)^23,††^	India	Population cross-sectional study	2863	51	Indian	1, 2, 4, 5	Age, T2DM, PXG, myopia
Doshi et al (2008)^34,‡‡^	USA	Population-based cross-sectional study	3583	55	Latino	3, 6	Age, IOP
Lam et al (2014)^21,§§^	Singapore	Population cross-sectional study	1704	63	Malay	4	Age, BMI, myopia, HbA1c, pulse pressure, HRT use
Shin et al (2018)^19,‖‖^	Korea	Population cross-sectional study	6860	56	Korean	1, 3, 4, 6	Age (factors 3 and 4); age; T2DM; HTN; myopia; BMI; OC use; PMH use (factors 1 and 6)
Lee et al (2019)^35,‖‖^	Korea	Population cross-sectional study	1798	60	Korean	2	Age, HTN, IOP

BMI = body mass index; HbA1c = glycated hemoglobin; HRT = hormone replacement therapy; HTN = hypertension; IOP = intraocular pressure; OC = oral contraceptive; PDR = proliferative diabetic retinopathy; PMH = postmenopausal hormone use; PXG = pseudoexfoliation glaucoma; T2DM = type 2 diabetes mellitus.

* The factors assessed are shown with 1 indicating age at menarche, 2 indicating parity, 3 indicating OC use, 4 indicating age at menopause, 5 indicating reproductive duration, and 6 indicating PMH use.

† Women’s Health Initiative Sight Examination.

‡ Covariables were assessed but not included in the final model owing to a lack of significance.

§ Nurses’ Health Study.

‖ Each individual analysis was adjusted for all listed covariates except the exposure of interest to that specific analysis.

¶ Mayo Clinic Cohort Study of Oophorectomy and Aging.

# Rotterdam Study.

** Blue Mountains Eye Study.

†† Aravind Comprehensive Eye Survey.

‡‡ Los Angeles Latino Eye Study.

§§ Singapore Malay Eye Study.

‖‖ Korean National Health and Nutrition Examination Survey.

**Table 2.** Characteristics of Studies Reporting an Association with Female Reproductive Factors and IOP Included in the Systematic Review*

Author (Yr)	Location	Design	Total Women Participants, n	Mean Age of Overall Study Population, Yrs	Predominant Ethnicity of Study Population	IOP Measurement Method	Adjustments
Controlled studies							
Treister and Mannor (1970)^25,†^	Israel	Prospective, controlled study	60	–	White	1	(Previous history of glaucoma)
Guaschino et al (2003)^36,†^	Italy	Prospective, controlled study (randomized)	80	60	White	2	(Menopausal <1 year; visual acuity >20/25; CTL use; medical contraindication to PMH; T2DM; HTN; glaucoma; thyroid diseases; any ophthalmic disease)
Affinito et al (2003)^27,†^	Italy	Prospective, controlled study (randomized)	50	53	White	1	(<1 year after menopause; medical contraindication to PMH use)
Abramov et al (2005)^37^	Israel	Prospective, controlled study	214	66	White	3	N/A (no statistical difference in age; CVD; BMI; HTN; hyperlipidemia; smoking habits between groups)
Vajaranant et al (2016)^8^	USA	Post hoc, ancillary analysis of data from randomized controlled trial	4347	72	White	1	Age, duration of PMH use, race, BMI, treatment adherence, lens status, diabetes, HTN, smoking, alcohol use
Uncontrolled studies							
Sator et al (1997)^29^	Austria	Prospective, uncontrolled study	25	56	White	1	(No recent use of PMH; β-blockers; clonidine; CAIs; no previous eye disease)
Toker et al (2003)^38^	Turkey	Retrospective, cohort study	62	52	White	1	Age, duration of amenorrhea (eye disease; T2DM; HTN; hypercholesterolemia; CVD; PVD)
Altintaş et al (2004)^4^	Turkey	Prospective study	44	–	White	1	Age (T2DM; CVD; HTN; ocular disease; CTL users; smokers)
Uncu et al (2006)^26^	Turkey	Prospective, uncontrolled study	30	50	White	1	(T2DM, HTN)
Verit et al (2007)^39^	Turkey	Cross-sectional study	77	50	White	1	(Previous PMH, topical eye medication, past ocular injury or infection, lid disorders, globe incongruity, ocular surface pathology, ocular surgery, any systemic or demyelinating disorder, T2DM, hormone malignancy, drug or alcohol use in past 12 months, cigarette use)
Deschênes et al (2010)^6^	Canada	Cross-sectional study	64	57	White	1	(BMI >30 kg/m^2^; current or former smoker; HTN; CVD; neurologic diseases; abnormal eye examination; vasoactive or anti-inflammatory medication use)
Tint et al (2010)^40^	UK	Prospective, cross-sectional study	263	62	White	3	Age, β-blocker use (intraocular disease, family history of glaucoma, refractive error >5 diopters)
Coksuer et al (2011)^28^	Turkey	Prospective study	34	52	White	1	(BMI >35 kg/m^2^; CAD; PVD; cerebrovascular disease; hyperlipidemia; T2DM; HTN; smoking; alcohol use; history of VTE; liver or renal disease
Na et al (2014)^41^	Korea	Population cross-sectional study	3968	63	Korean	1	Age, T2DM, HTN, high cholesterol levels, or high LDL cholesterol (systemic inflammatory disorder; infectious disease)
Özcan et al (2017)^30^	Turkey	Cross-sectional study	137	50	White	1	N/A

BMI = body mass index; CAD = coronary artery disease; CAI = carbonic anhydrase inhibitor; CTL = contact lens; CVD = cardiovascular disease; HTN = hypertension; IOP = intraocular pressure; LDL = low-density lipoprotein; N/A = not available; PMH = postmenopausal hormone use; PVD = peripheral vascular disease; T2DM = type 2 diabetes mellitus; VTE = venous thrombus embolism.

* The IOP measurement methods are shown with 1 indicating Goldmann Applanation Tonometry, 2 indicating noncontact tonometry, and 3 indicating Perkins Applanation Tonometry. Exclusions in each study are indicated in parentheses.

† Women’s Health Initiative Sight Examination.

**Table 3.** Association between Age at Menarche and Open-Angle Glaucoma Risk in Included Studies*

Author (Yr)	Subjects, N	Cases, N	Age at Menarche	Multivariable Adjusted Effect Estimate (95% CI)*
Cohort studies				
Pasquale and Kang (2011)^15^	288 270^†^	172	< 12	Ref
	344 678	205	12	0.9 (0.8–1.1)
	400 956	241	13	0.9 (0.8–1.2)
	254 296	190	> 13	1.1 (0.9–1.3)
Cross-sectional studies				
Hulsman et al (2001)^22^	3078	78	N/A	1.1 (0.9–1.2) ^‡^
Lee et al (2003)^24^	691	14	< 13	Ref
	441	10	13	2.1 (1.1–3.8)^§^
	824	41	> 14	2.0 (1.0–3.9)
Nirmalan et al (2004)^23^	662	5	< 13	Ref
	2135	15	> 14	1.0 (0.3–2.9)
Shin et al (2018)^19^	6463	397	≤ 12	1.0 (0.4–2.7)
			13–16	Ref
			≥ 17	0.8 (0.9–2.7)

CI = confidence interval; N/A = not available; Ref = reference.

* Odds ratio, hazard ratio, or relative risk effect estimate from the most fully adjusted model.

† Person-years of follow-up.

‡ Effect estimate for open-angle glaucoma for each additional year later of age at menarche.

§ Statistically significant at a *P* value < 0.05.

**Table 4.** Association between Oral Contraceptive Use and Open-Angle Glaucoma Risk in Included Studies*

Author (Yr)	Total Subjects, N	Cases, N	Multivariable Effect Estimate (95% CI)^†^	Duration of Use
Cohort studies				
Pasquale and Kang (2011)^15^	619 356^‡^	333	1.1 (1.0–1.3)	Ever
	214 196^‡^	135	1.3 (1.02–1.53)^§^	>5 years
Cross-sectional studies				
Lee et al (2003)^24^	649	72	0.4 (0.2–1.2)	Ever
Doshi et al (2008)^34^	716	18	0.8 (0.5–1.5)	Ever
Shin et al (2018)^19^	1208	397	0.8 (0.5–1.1)	Ever
			0.9 (0.4–2.1)	>3 years

CI = confidence interval.

* Pasquale and Kang (2011)^15^ is repeated twice in this table, as the study conducted analyses for both ever using an OC and duration of OC use > 5 years.

† Odds ratio, hazard ratio, or relative risk effect estimate from the most fully adjusted model.

‡ Person-years of follow-up.

§ Statistically significant at a *P* value < 0.05.

**Table 5.** Association between Parity and Open-Angle Glaucoma Risk in Included Studies

Author (Yr)	Parity, N	Subjects, N	Cases, N	Multivariable adjusted effect estimate (95% CI)*
Cohort studies				
Pasquale and Kang (2011)^15^	0	69 918^†^	37	0.9 (0.6–1.2)
	1–2	448 136	254	Ref
	3	362 274	235	1.1 (0.9–1.3)
	≥ 4	400 590	280	1.0 (0.8–1.2)
Cross-sectional studies				
Lee et al (2003)^24^	0	400	13	Ref
	1–2	746	18	0.8 (0.4–1.7)
	3–4	643	26	1.6 (0.8–3.2)
	≥ 5	193	11	2.5 (1.1–6.1)^‡^
Nirmalan et al (2004)^23^	0	167	1	Ref
	1–2	519	0	—
	3–4	896	5	1.3 (0.2–9.6)
	≥ 5	1253	14	1.5 (0.2–12)
Lee et al (2019)^43^	0	8	1	4.9 (0.5–50)
	1	113	5	1.7 (0.5–6.2)
	2	729	29	Ref
	3–4	687	51	2.1 (1.1–3.9)^‡^
	≥ 5	261	30	2.1 (0.9–4.8)
Shin et al (2018)^19^	—	6463	397	1.0 (0.9–1.1)^§^

CI = confidence interval; Ref = reference.

* Odds ratio, hazard ratio, or relative risk effect estimate from the most fully adjusted model.

† Person-years of follow-up.

‡ Statistically significant at a *P* value < 0.05.

§ Effect estimate for open-angle glaucoma for each additional pregnancy.

**Table 6.** The Association between Age at Menopause and OAG Risk in Included Studies

Author (Yr)	Study Design	Age at Menopause	Number at Risk, N	Cases, N	Multivariable Effect Estimate (95% CI)*
Age at menopause (secondary to surgical causes)					
Vajaranant et al (2014)^16^ [OAG cases only]^†^	Age-matched, retrospective cohort study	< 43	9120^‡^	11	1.0 (0.5–1.9)
		43–48	17 206	16	1.0 (0.6–1.8)
		≥ 48		16	0.7 (0.4–1.2)
Vajaranant et al (2014)^16^ [all glaucoma diagnoses]^†^		< 43	—	97	1.6 (1.5–2.2)^§^
		43–48		47	1.1 (0.8–1.6)
		≥ 48		50	0.8 (0.6–1.2)
Age at menopause (combined natural and surgical causes)					
Pasquale et al (2007)^14^	Prospective cohort study	≥ 54	128 101^‡^	63	0.8 (0.6–1.2)
		50–54	212 006	134	Ref
		45–49	251 205	178	1.0 (0.8–1.3)
		< 45	69 382	44	0.9 (0.6–1.1)
Pasquale et al (2007)^14^ [secondary analysis of subjects aged ≥ 65]		≥ 54	—	222	0.5 (0.3–0.9)^§^
		50–54			Ref
Hulsman et al (2001)^22^	Population cross-sectional study	≥ 50	3078	—	Ref
		45–49		78	1.1 (0.7–1.9)
		< 45		—	1.8 (1.0–3.0)
Lee et al (2003)^24^	Population cross-sectional study	≥ 50	788	—	Ref
		45–49	442	72	1.2 (0.6–2.3)
		<45	340	—	1.3 (0.7–2.6)
Lam et al (2014)^21^	Population cross-sectional study	≥ 53	266	7	Ref
		< 53	870	43	3.4 (1.2–9.8)^§^
Shin et al (2018)^19^	Population cross-sectional study	≥ 45	—	—	Ref
		< 45	918	39	1.6 (0.9–2.7)
Age at natural menopause only					
Pasquale et al (2007)^14^	Prospective cohort study	≥ 54		315	0.9 (0.6–1.2)
		50–54	—		Ref
		< 45	—	41	0.6 (0.4–1.1)
Hulsman et al (2001)^22^	Population cross-sectional study	≥ 50	2027	19	Ref
		45–49			1.1 (0.7–2.0)
		< 45	—	18	2.6 (1.5–4.8)^§^
Lee et al (2003)^24^	Population cross-sectional study	≥ 50	719	N/A	Ref
		45–49	346		1.2 (0.6–2.5)
		< 45	164		1.7 (0.7–3.8)
Nirmalan et al (2004)^23^	Population cross-sectional study	≥ 50	125	1	Ref 1.1(0.9–14.5)
		45–49	603	8	
		< 45	2071	11	1.1(0.1–16.0)
Lam et al (2014)^21^	Population cross-sectional study	≥ 53	—	7	Ref
		< 53	97	41	3.5 (1.2–10.1)^§^
Shin et al (2018)^19^	Population cross-sectional study	≥ 45	—	—	Ref
		< 45			2.3 (1.2–4.5)^§^

CI = confidence interval; N/A = not available; OAG = open-angle glaucoma; Ref = reference.

* Effect estimate from the most fully adjusted model (odds ratio of primary OAG for each additional year of age at menopause).

† For these analyses, the reference group is age-matched women who underwent a hysterectomy without oophorectomy.

‡ Person-years of follow-up.

§ Statistically significant at a *P* value < 0.05.

**Table 7.** The Association between Age at Menopause and Intraocular Pressure > 21 mmHg in Included Studies

Author (Yr)	Study Design	Age at Menopause	OAG Cases, N	Multivariable RR (95% CI)*
Hulsman et al (2001)^22^	Population cross-sectional study	≥ 50	172	Ref
		45–49	62	0.8 (0.6–1.1)
		< 45	42	1.2 (0.8–1.8)
Pasquale et al (2007)^14^	Prospective cohort study	≥ 54	41	0.8 (0.6–e1.3)
		50–54	91	Ref
		45–49	115	1.1 (0.8–1.4)
		< 45	28	0.9(0.6–1.2)

CI = confidence interval; OAG = open-angle glaucoma; Ref = reference; RR = relative risk.

* Effect estimate from the most fully adjusted model.

**Table 8.** The Association between Reproductive Duration and OAG Risk in Included Studies

Author (Yr)	Study Design	Reproductive Duration, Yrs	Number at Risk, N	Cases, N	Multivariable OR (95% CI)*	Mean Duration of Reproductive Years
Hulsman et al (2001)^22^	Population cross-sectional	N/A	3078	78	0.95 (0.90–0.99)^†,‡^	36.1
Lee et al (2003)^24^	Population cross-sectional	≥ 40	297	72	Ref	34.6
		35–39	629		1.4 (0.6–3.3)	
		30–34	346		1.7 (0.7–4.3)	
		< 30	287		1.6 (0.6–4.3)	
Nirmalan et al (2004)^23^	Population cross-sectional	≥ 35	187	1	Ref	28.4
		30–34	876	10	2.2 (0.2–27.1)	
		< 30	1734	9	1.6 (0.1–23.0)	
Pasquale and Kang (2011)^15^	Prospective cohort study	≥ 40	15 952^‡^	115	0.9 (0.7–1.3)	N/A
		39–40	189 380	146	Ref	
		36–38	121 454	106	0.9 (0.7–1.2)	
		< 36	117 167	100	0.9 (0.7–1.2)	

CI = confidence interval; N/A = not available; OR = odds ratio; POAG = primary open-angle glaucoma; Ref = reference.

* Effect estimate from the most fully adjusted model.

† Statistically significant at a *P* value < 0.05.

‡ Odds ratio for POAG for each additional year of reproductive duration.

**Table 9.** The Association between Type of PMH Used and Risk of Open-Angle Glaucoma in Randomized Controlled Trials and Cohort Studies*

Author (Yr)	Study Design	Mean Age	Total Subjects, N	Cases, N	Duration, Yrs	Multivariable Effect Estimate (95% CI)^†^	Dose, mg/day
Estrogen-only PMH							
Vajaranant et al (2018)^17,‡^	Post hoc, ancillary analysis of data from randomized controlled trial	69	3510	319	4.4	1.01 (0.8–1.6)	0.625 CEE
Pasquale et al (2007)^14^	Prospective cohort study	60	103 460^§^	66	—	1.0 (0.73–1.35)	N/A
Newman-Casey et al (2014)^18^	Retrospective cohort study using record linkage or historic data	66	59 847	1027	2.1	0.996 (0.993–0.999)^‖^	N/A
Combined estrogen-and-progesterone PMH							
Vajaranant et al (2018)^17^	Post hoc, ancillary analysis of data from randomized controlled trial	69	4592	357	4.4	1.05 (0.85–1.29)	0.625 CEE + 2.5 MPA
Pasquale et al (2007)^14^	Prospective cohort study	60	79 426^§^	46	—	0.87 (0.61–1.22)	N/A
Newman-Casey et al (2014)^18^	Retrospective cohort study using record linkage or historic data	66	15 288	296	1.9	1.0 (0.98–1.0)	N/A

CEE = conjugated equine estrogen; CI = confidence interval; MPA = methylprogesterone; N/A = not available; PMH = postmenopausal hormone.

* Pasquale et al (2007),^14^ Newman-Casey et al (2014),^18^ and Vajaranant et al (2018)^17^ are repeated twice in this table, as they provide independent analyses for different types of PMH.

† Effect estimate from the most fully adjusted model.

‡ Randomized controlled trial.

§ Person-years of follow-up.

‖ Statistically significant at a *P* value < 0.05.

**Table 10.** The Association between PMH Use and Odds of Open-Angle Glaucoma in Cross-sectional Studies

Author (Yr)	Study Design	Mean Age	Total Subjects, N	Cases, N	PMH Users, N	OR (95% CI)*	Duration of Use, Mos
Hulsman et al (2001)^22^	Population cross-sectional study	68	3078	93	188	0.54 (0.17–1.74)	30
Lee et al (2003)^24^	Population cross-sectional study	62	2072	72	557	0.50 (0.20–1.20)	N/A
Doshi et al (2008)^34^	Population cross-sectional study	55	6142	291	553	0.81 (0.47–1.40)	N/A

Shin et al (2018)^19^	Population cross-sectional study	56	6860	397	745	0.90 (0.58–1.39)	N/A

CI = confidence interval; N/A = not available; OR = odds ratio; PMH = postmenopausal hormone.

* Odds ratio from the most fully adjusted model for primary open-angle glaucoma in PMH users compared with nonusers.

**Table 11.** Mean Difference in IOP between PMH Users and Nonusers (Controlled Studies)*

Author (Yr)	Study Design	PMH Users, N	PMH Nonuser Controls, N	IOP in PMH Users, mmHg	IOP in Nonuser Controls, mmHg	Mean Difference in IOP, mmHg (95% CI)	Average Duration of Use, Mos
Estrogen-only PMH							
Vajaranant et al (2016)^8,†,‡^	Secondary analysis of data from randomized controlled trial	808	860	15.4 3.2	15.8 3.3	−0.5^§^(−0.8 to −0.1)	60
Treister and Mannor (1970)^25^	Prospective controlled study	15	15	—	—	−2.0^§^(−2.4 to −1.6)	6
Estrogen-and-progesterone PMH							
Treister and Mannor (1970)^25^	Prospective controlled study	15	15	—	—	−1.8^§^ (−2.2 to −1.4)	6
Guaschino et al (2003)^36,‖^	Prospective randomized controlled trial	40	40	14.8 ± 3.2	14.9 ± 4.3	−0.1	12
Affinito et al (2003)^27,‖^	Prospective controlled study (randomized)	25	25	14.1 ± 2.0	16.6 ± 2.4	−2.5^§^	6
Abramov et al (2005)^37,†,‖^	Prospective controlled study	107	107	15.2 ± 0.4	15.5 ± 0.4	−0.3	84
Vajaranant et al (2016)^8,†,‡^	Post hoc, ancillary analysis of data from randomized controlled trial	1397	1282	15.6 ± 3.0	15.7 ± 3.1	−0.1 (−0.4 to 0.1)	60

CI = confidence interval; IOP = intraocular pressure; PMH = postmenopausal hormone.

* Treister and Mannor (1970)^25^ and Vajaranant et al (2016)^8^ are repeated twice in this table, as these studies reported independent analyses based on the type of PMH used.

† Data were reported separately for each eye, and data from the right eye are presented in this table.

‡ Randomized controlled trial.

§ Significant at a *P* value < 0.05.

‖ The 95% CI for the mean difference between groups was not reported in the referenced study, and the mean difference was computed by authors of this review (K.M.M., S.Y.L.C).

**Table 12.** Difference in IOP between PMH Users and Nonusers (Uncontrolled Studies)

Author (Yr)	Study Design	PMH Users, N	PMH Nonusers, N	IOP in PMH Users, mmHg	IOP in Nonusers, mmHg	Mean Difference IOP, mmHg	Average Duration of Use, Mos
Estrogen + Progesterone PMH							
Toker et al (2003)^38^	Retrospective cohort study	30	32	13.3 ± 2.3	13.6 ± 2.5	−0.3	48
Tint et al (2010)^40^	Prospective cross-sectional study	91	172	11.9 ± 2.7	13.2 ± 2.9	−1.3*	—
Verit et al (2007)^39^	Cross-sectional study	40	37	13.7 ± 3.4	13.8 ± 4.0	−0.1	6
Deschênes et al (2010)^6^	Cross-sectional study	35	29	14.1 ± 2.2	14.5 ± 1.7	−0.4	96
Na et al (2014)^41^	Population cross-sectional study	578	3390	14.8 ± 0.1	14.4 ± 0.3	0.4*	—
Özcan et al (2017)^30^	Cross-sectional study	61	76	13.9 ± 1.5	14.4 ± 1.4	−0.5	—

IOP = intraocular pressure; PMH = postmenopausal hormone.

* Statistically significant at a *P* value <0.05.

**Table 13.** Difference in IOP within Participants, Before and After PMH Use (Before and After Uncontrolled Studies)*

Author (Yr)	Study Design	PMH Users, N	IOP at Baseline, mmHg	IOP at Study End, mmHg	Mean Change in IOP, mmHg	Duration of Follow-up, Mos
Estrogen-only PMH						
Uncu et al (2006)^26^	Prospective uncontrolled study	10	14.6 ± 0.8	12.6 ± 0.7	−2.0^†^	12
Combined estrogen-and-progesterone PMH						
Uncu et al (2006)^26^	Prospective uncontrolled study	38	13.8 ± 0.4	14.1 ± 0.3	0.3	12
Altintaş et al (2004)^4^	Prospective study	15	16.1 ± 2.2	12.7 ± 1.7	−3.8^†^	2
Coksuer et al (2011)^28^	Prospective study	34	14.1 ± 2.8	13.4 ± 2.7	−0.7^†^	6
Sator et al (1997)^29,‡^	Prospective uncontrolled study	25	15.3 ± 2.3	14.0 ± 1.9	−1.3^†^	3

IOP = intraocular pressure; PMH = postmenopausal hormone.

* Uncu et al (2006)^26^ is repeated twice in this table, as this study reported independent analyses using different types of PMH.

† Statistically significant at a *P* value < 0.05.

‡ Data were reported separately for each eye, and data from the right eye are presented in this table.

## Abbreviations and Acronyms:

CI: confidence interval
HR: hazard ratio
IOP: intraocular pressure
NHS: Nurses’ Health Study
OAG: open-angle glaucoma
OC: oral contraceptive
OR: odds ratio
PMH: postmenopausal hormone
POAG: primary open-angle glaucoma
RCT: randomized controlled trial
RGC: retinal ganglion cell
SES: socioeconomic status
