# Supplementary material for: The Association of Female Reproductive Factors with Glaucoma and Related Traits
Source: Ophthalmol Glaucoma. Author manuscript; Available in PMC 2023 Mar 29. (PMC10051419; doi:10.1016/j.ogla.2022.06.003)
Supplement: Table S2 [file NIHMS1876580-supplement-Table_S2.pdf]

Supplemental Table A – Case definition of open-angle glaucoma (OAG) / primary open-angle glaucoma (POAG) in included studies

Study	Direct Examination	Review of Previous Examination / Record Linkage	VF Assess.	ON Assess.	Angle Assess.	Exclusion of / adjustment for other OAGs	OAG diagnosis independent of IOP
Huslman et al (2001)	Yes	No	Yes	Yes	No	No	Yes
Lee et al (2003)	Yes	No	Yes	Yes	Yes	Yes	Yes
Nirmalan et al (2004)	Yes	No	Yes	Yes	No	Yes	Yes
Shin et al (2018)	Yes	No	Yes	Yes	No	N/A	Yes*
Lee et al (2019)	Yes	No	Yes	Yes	No	N/A	Yes*
Doshi et al (2007)	Yes	No	Yes	Yes	Yes	N/A	Yes
Lam et al (2014)	Yes	No	Yes	Yes	No	N/A	Yes*
Vajaranant et al (2014)	No	Yes	-	-	-	Yes	-
Newman-Casey et al (2014)	No	Yes	-	-	-	Yes	-
Vajaranant et al (2018)	No	Yes	-	-	-	Yes	-
Pasquale et al (2011)	No	Yes	Yes	No	Yes	Yes	Yes
Pasquale et al (2007)	No	Yes	Yes	No	Yes	Yes	Yes

*case ascertainment based on ISGEO criteria in which category III includes IOP (>99.5th percentile) as a component of the definition of POAG

VF = visual fields; ONH = optic nerve; IOP = intraocular pressure
