# Supplementary material for: The Association of Female Reproductive Factors with Glaucoma and Related Traits
Source: Ophthalmol Glaucoma. Author manuscript; Available in PMC 2023 Mar 29. (PMC10051419; doi:10.1016/j.ogla.2022.06.003)
Supplement: Table S1 [file NIHMS1876580-supplement-Table_S1.pdf]

Suppl Table 1. Characteristics of studies reporting an association between a female reproductive factor and glaucoma or related traits in human subjects but not meeting criteria for inclusion in the systematic review

Author (year)	Location	Design	Age (years)	Size (cases)	Result / effect estimate (95% CI)	Exclusion
Agrawal (2018)[80]	Nepal	CS	15-35	120	IOP was lower in second trimester of pregnancy compared to first trimester (p<0.001), and from first to second trimester (P<0.001). The difference between second and third trimester was not significant (P=0.09).	1
Akar (2004)[81]	Turkey	CS	25	38	IOP was no different between follicular, ovulatory phases and luteal phases of menstrual cycle (p=0.34).	1
Atilla (2001)[82]	Turkey	CS	50	20/20	No significant difference in flow velocity, vascular resistance or pulsatility indices between postmenopausal PMH and non-PMH users (P>0.05)	2
Bahadir Kilavuzoglu (2017)[83]	Turkey	CS	32	50/50	IOP was negatively correlated with gestational week of pregnancy (r = -0.32, P = 0.02). IOP was significantly lower in the pregnant group than in the non-pregnant group (16.2mmHg±2.3 vs 18.4mmHg±-3.4, P<0.001).	1
Battaglia (2004)[57]	Italy	CC	55	8/15	PMH administration in postmenopausal women was associated with improved plasma viscosity and ophthalmic artery doppler parameters (-43% vs -28%; P=0.001)	2
Ebeigbe (2011)[84]	Nigeria	CH	20-35	100	Advancing pregnancy trimester was significantly associated with lower IOP. IOP was 14.7mmHg ±2.2, 13.2mmHg ±2.0, 11.0mmHg ±1.3 (difference between groups P<0.001).	1
Goldich (2014)[85]	Israel	CC	29	60/60	Goldmann-correlated and corneal-compensated IOP (IOPcc) were significantly lower in the pregnant group compared to the non-pregnant group (10.9mmHg vs 13.0mmHg, P<0.001 for Goldmann; 11.0mmHg vs 13.1mmHg P<0.001 for IOPcc)	1
Green (1984)[86]	UK	CS	31	3	No correlation identified between IOP and occurrence/phase of menstrual cycle (no statistical analyses)	1
Harris-Yitzhak (2000)[5]	USA	CS	56	16/16	Postmenopausal women receiving PMH had lower resistance indices in the ophthalmic artery compared to post-menopausal women not receiving PMH (P<0.001)	2
Horven (1974)[87]	Norway	CH	25	55	IOP was lower during pregnancy and during the first two months after delivery (no statistical analyses)	**1**
Kang (2018)[88]	USA	CC	61/63	189	Plasma sex hormone levels of estrone and estradiol in post-menopausal women were not associated with POAG risk	1
Kang (2011)[11]	USA	CC	>40/	374 / 1 085	Among women with age at menarche <13 years, compared with the CC homozygotes of the rs3918188 tagging SNP, the wild-type AA homozygotes were at significantly reduced risk of POAG (RR=0.31, 95% CI=0.16, 0.59). No significant interactions between *NOS3* SNPs and OC use were identified	2

Kang (2010)[7]	USA	CC	>/ 40	527 /1 543	Among women, 4 of the 5 assessed NOS3 SNPs showed significant interactions with PMH use in relation to POAG with IOP > 21mmH. In women with the TT genotype, PMH use was inversely associated with POAG risk (RR = 0.41, 95%CI 0.22-0.76)	2
Lee (2019)[42]	Australia	CH	20	484 (10)	Women with parity >1 had larger vertical neuroretinal rim widths than nulliparous women (p<0.001)	1
Naderan (2017)[89]	Iran	CH	26	70/70	No significant difference in IOPcc was identified between females followed from pre-pregnancy through to the third trimester and 12 months post-partum (P>0.05)	1
Park (2017)[90]	Korea	CS	64	4 057	Later age at first delivery (over 27) was associated with decreased risk of POAG among post-menopausal women (OR = 0.31, 95%CI 0.13,0.71 P = 0.006)	1
Pasquale (2017)[47]	USA	CC	50	21 060 / 29 110	Genetic risk score predicting 4.8% of age at natural menopause variation was not associated with POAG (OR = 1.002; 95CI 0.998, 1.007 P =0.28)	1
Philips (1985)[91]	UK	CS	28	44	Mean IOP of non-pregnant patients did not differ significantly from women in first trimester of pregnancy. Mean IOP was lower by 2.2mmHg(2.2SD), P<0.05 in third trimester compared to first trimester.	1
Qureshi (1996)[92]	Pakistan	CS	N/A	200	Third trimester of pregnancy was associated with lower IOP compared to non-pregnant controls (13.6mmHg± 1.9 vs 15.6mmHg±1.5, P<0.001).	1
Sator (1998)[93]	Austria	C	56	1	During HRT use IOP was reduced by from 20mmHg 15mmHg over 12 weeks (no statistical analysis)	2
Saylik (2014)[94]	Turkey	CS	N/A	120	Mean IOP in pregnant women carrying two fetuses was lower compared to pregnant women carrying a single fetus (11.4mmHg ±1.2 vs 13.1mmHg ±0.8, p=0.011) and lower than in non-pregnant women (9.8mmHg ±1.36 vs 15.08mmHg ±1.18, P=0.011)	1
Siuw (2018)[95]	Malaysia	CS	52	49/49	Mean IOP in postmenopausal women was significantly higher than mean IOP in premenopausal women (15.3mmHg ± 3.0 vs. 14.1mmHg ± 2.7, P = 0.04).	1
Souza-Junior (2015)[96]	Brazil	CS	27	45	The superior RNFL around the optic nerve was significantly thicker in patients with polycystic ovarian syndrome (128 µm ±18) vs healthy controls (120 µm±15.1), P=0.03	De Souza-Junior (2015)
Sundaram (2016)[97]	India	CH	25	100	Lower IOP was found across advancing trimester of pregnancy (13.9mmHg, ±2.1; 12.6mmHg ±1.5; 11.1mmHg ± 1.03, in first, second, third trimester of pregnancy respectively, P<0.001)	1
Wang (2016)[20]	USA	CS	>/ 40	3 406 (231)	Greater than 3 years of OC use was associated with higher odds (OR = 1.94, 95%CI 1.22-3.07) of self-reported glaucoma or ocular hypertension	2

1-Study exposure not relevant to review / exposure did not meet pre-specified inclusion criteria ; 2- study outcome not relevant to review/ outcome did not meet pre-specified inclusion criteria; CS, cross-sectional; CC, case-control; CH, cohort; C, case study, OR, odds ratio; CI, confidence interval; IOP, intraocular pressure; IOPcc, corneal-compensated intraocular pressure; OHT, ocular hypertension; OAG, open-angle glaucoma; mmHg, millimeters of mercury; RNFL, retinal nerve fiber layer; HRT, hormone-replacement therapy; PMH, post-menopausal hormone; OC, oral contraceptive; SNP, single-nucleotide polymorphism; NOS3, nitric oxide synthase 3
