# Supplementary material for: The Association of Female Reproductive Factors with Glaucoma and Related Traits
Source: Ophthalmol Glaucoma. Author manuscript; Available in PMC 2023 Mar 29. (PMC10051419; doi:10.1016/j.ogla.2022.06.003)
Supplement: Appendix SA1 [file NIHMS1876580-supplement-Appendix_SA1.pdf]

Appendix A – Search Strategy for the EMBASE and Medline database using the Ovid interface

Database: Embase

----

1 glaucoma/ or glaucomatous optic neuropathy/ or intraocular hypertension/ or low tension glaucoma/ or open angle glaucoma/ (74578)
2 glaucoma.mp. (91384)
3 glaucom*.mp. (92299)
4 (POAG or OAG).mp. (7141)
5 (IOP or intraocular pressure or intra-ocular pressure).mp. (76411)
6 intraocular pressure/ (55738)
7 trabecular meshwork.mp. (6718)
8 aqueous humor.mp. or aqueous humor/ (15337)
9 1 or 2 or 3 or 4 or 5 or 6 or 7 or 8 (143368)
10 estrogen/ (104350)
11 estrogen*.mp. (266702)
12 oestrogen*.mp. (25999)
13 (ovary or ovari* or uterus).mp. (542880)
14 (progest* or progesterone*).mp. (162536)
15 progesterone/ (88571)
16 menopause.mp. or menopause/ or early menopause/ (67596)
17 menstruation/ (19091)
18 (menstruation or menstrua*).mp. [mp=title, abstract, heading word, drug trade name, original title, device manufacturer, drug manufacturer, device trade name, keyword, floating subheading word, candidate term word] (87656)
19 estrogen therapy/ or hormone substitution/ (57623)
20 (estrogen replacement or estrogen therapy or hormone therapy or hormone replacement therapy).mp. (79043)
21 hormone replacement therapy.mp. or hormone substitution/ (42892)
22 hormonal contraception/ or contraception/ or contraception.mp. or oral contraception/ (70787)
23 (oral contracepti* or birth control).mp. (60873)
24 pregnancy/ or pregnancy.mp. (862775)
25 pregnant.mp. (255769)
26 parturition.mp. or birth/ (31284)
27 birth.mp. (445843)
28 parity/ (35894)
29 parity.mp. (52321)
30 gravidity.mp. (3781)
31 gravid*.mp. (24208)
32 menarche.mp. or menarche/ (15001)
33 10 or 11 or 12 or 13 or 14 or 15 or 16 or 17 or 18 or 19 or 20 or 21 or 22 or 23 or 24 or 25 or 26 or 27 or 28 or 29 or 30 or 31 or 32 (2038539)
34 9 and 33 (2651)
****

Database: Ovid MEDLINE(R) and Epub Ahead of Print, In-Process & Other Non-Indexed Citations and Daily <1946 to September 15, 2020>
----
1 glaucoma/ or glaucoma, open-angle/ or low tension glaucoma/ (49267)
2 (glaucoma or glaucom*).mp. (71587)
3 (POAG or OAG).mp. (5519)
4 Intraocular Pressure/ (37815)
5 (intraocular pressure or IOP).mp. (51891)
6 trabecular meshwork.mp. or Trabecular Meshwork/ (6005)
7 Aqueous Humor/ or aqueous hum*.mp. (14736)
8 Estrogens/ (57684)
9 estrogen.mp. (149978)
10 estradiol.mp. or Estradiol/ (124088)
11 Progesterone/ or progesterone.mp. (109764)
12 Menopause, Premature/ or Menopause/ or menopause.mp. (45057)
13 menstruation.mp. or Menstruation/ (29701)
14 Menstrual Cycle/ (13262)
15 menstrua*.mp. (64533)
16 (estrogen replacement therapy or estrogen replacement or oestrogen replacement therapy or oestrogen replacement).mp. (17685)
17 Hormone Replacement Therapy/ or Estrogen Replacement Therapy/ or hormone replacement.mp. (32648)
18 PMH.mp. (10506)
19 progestin.mp. (11663)
20 Progestins/ (10574)
21 (contraceptive or contraception or oral contracepti* or OCP).mp. (85085)
22 oral contraceptive.mp. or Contraceptives, Oral/ (25869)
23 birth control.mp. (5288)
24 Pregnancy/ or pregnancy.mp. (967879)
25 pregnant.mp. (187079)
26 parturition.mp. or Parturition/ (22915)
27 birth.mp. (339357)
28 parity.mp. or Parity/ (49712)
29 gravidity.mp. or Gravidity/ (3685)
30 gravid*.mp. (23513)
31 menarche.mp. or Menarche/ (9988)
32 menarch*.mp. (10262)
33 1 or 2 or 3 or 4 or 5 or 6 or 7 (103255)
34 8 or 9 or 10 or 11 or 12 or 13 or 14 or 15 or 16 or 17 or 18 or 19 or 20 or 21 or 22 or 23 or 24 or 25 or 26 or 27 or 28 or 29 or 30 or 31 or 32 (1556658)
35 33 and 34 (1865)
****

Database: Cochrane Central Register of Controlled Trials
[- to Sept 15 2020]

1. MeSH descriptor: [Glaucoma] explode all trees
2. POAG
3. OAG
4. Intraocular pressure
5. IOP
6. Ocular hypertension
7. Ocular pressure
8. MeSH descriptor: [Estrogens] explode all trees
9. Progesterone
10. Reproductive hormone*
11. MeSH descriptor: [Menarche] explode all trees
12. Menarch*
13. MeSH descriptor: [Parity] explode all trees
14. *parity
15. MeSH descriptor: [contraception] explode all trees
16. Oral contraceptive
17. Birth control
18. Reproductive years
19. Reproductive duration
20. Endogenous estrogen
21. Oral contrapcet*
22. MeSH descriptor: [Menopause] explode all trees
23. Menopaus*
24. MeSH descriptor: [Estrogen Replacement Therapy] explode all trees
25. #1 OR #2 OR #3 OR #4 OR #5 OR #6 OR #7
26. #8 OR #9 OR #10 OR #11 OR #12 OR #13 OR #14 OR #15 OR #16 OR #17 OR #18 OR #19 OR #20 OR #21 OR #22 OR #23 OR #24 OR #25
27. #25 AND #26 [211 Cochrane Reviews identified; 86 trials; 8 Cochrane protocols]

Database: Google Scholar
(glaucoma OR primary open angle glaucoma OR POAG OR OAG OR IOP OR intraocular pressure OR ocular hypertension OR ocular pressure) AND (reproductive hormones OR menarche OR parity OR oral contraceptive OR menopause OR post-menopausal hormone OR hormone replacement therapy) [1300 results]
